# Program Access, Depressive Symptoms, and Medical Errors Among Resident Physicians With Disability

**DOI:** 10.1001/jamanetworkopen.2021.41511

**Published:** 2021-12-30

**Authors:** Lisa M. Meeks, Karina Pereira-Lima, Elena Frank, Erene Stergiopoulos, Katherine E.T. Ross, Srijan Sen

**Affiliations:** 1 Department of Family Medicine, University of Michigan Medical School, Ann Arbor; 2 Department of Psychiatry, University of Michigan Medical School, Ann Arbor; 3 Michigan Neuroscience Institute, University of Michigan Medical School, Ann Arbor; 4 Department of Psychiatry, University of Toronto, Toronto, Ontario, Canada; 5 Department of Psychology, University of Michigan, Ann Arbor; 6 Eisenberg Family Depression Center, University of Michigan Medical School, Ann Arbor

## Abstract

This cohort study uses data from a survey of US medical interns to assess the prevalence of self-reported disability and program accommodations and the association between accommodations, depressive symptoms, and self-reported medical errors among resident physicians.

## Introduction

Increases in the number of medical students reporting disability,^1^ coupled with disability-focused Accreditation Council for Graduate Medical Education (ACGME) regulations,^2^ have sparked a growing interest in disability for residency stakeholders. Although prevalence and characteristics of disability in medical student^1^ and physician^3^ populations are established, concurrent data for residents is lacking. Addressing this knowledge gap, we assessed the prevalence of self-reported disabilities, program access (ie, access needs self-reported as met via not needing accommodations or receiving accommodations), and the association between program access, depressive symptoms, and self-reported medical errors in a large, multispecialty cohort of US intern physicians.

## Methods

Interns from 282 institutions across 22 specialties who enrolled in the longitudinal Intern Health Study^4^ in 2020 completed an online baseline survey 2 months prior to beginning their internships. The survey assessed depressive symptoms using the Patient Health Questionnaire-9 (PHQ-9). In an end-of-year (ie, the 12th month of the internship) follow-up survey, participants completed the PHQ-9, a question about medical errors,^4^ and disability questions that directly mirrored those in the Association of American Medical Colleges Graduation Questionnaire,^5^ which reports whether a resident has a disability (yes, no, or “I don’t know”), category of disability, and program access (eMethods in the Supplement). Only residents with clear responses (ie, yes or no) were included in the analysis. All participants provided written informed consent and were compensated $80 to $130. This cohort study was approved by the University of Michigan institutional review board and followed the Strengthening the Reporting of Observational Studies in Epidemiology (STROBE) reporting guideline. Data were analyzed September 2021.

Mann-Whitney or χ^2^ tests were used to examine differences in demographic (age, gender, sexual orientation, and race and ethnicity) and academic characteristics (specialty and residency institution type) by reported disability status. Differences in self-reported medical errors and changes in PHQ-9 depressive symptoms (ie, the difference between end-of-year and baseline PHQ-9 scores) based on program access and reported disability status were examined using χ^2^ and Kruskal-Wallis tests, followed by Dunn-Bonferroni post hoc for pairwise comparisons. Program access was defined as met for residents with disabilities (RWD) who reported receiving accommodations or not needing accommodations. A 2-sided *P* < .05 was considered significant. Analyses were conducted using SPSS version 21 (IBM Corp).

## Results

Overall, 2472 (51.8%) invited residents participated in the study. Of those, 1273 residents (51.4%; 706 women [55.5%], median [IQR] age 27.0 [26.0-29.0] years) responded yes or no to the disability questions and were included in the analysis (30 residents [2.3%] responded “I don’t know” and were excluded) (Table 1). A total of 96 participants (7.5%) self-reported a disability. Most RWD self-reported having program access through accommodations (31 respondents [32.3%]) or that accommodations were not required for access (51 respondents [53.1%]).

**Table 1.** Demographic, Academic, and Disability Characteristics of Study Participants

Characteristic	Residents, No. (%)		*P* value ^a^
	With reported disabilities (n = 96)	Without reported disabilities (n = 1177)	
Overall prevalence	96 (7.5)	1177 (92.5)	NA
Demographic characteristics			
Gender			
Women	54 (53.6)	652 (55.4)	.87
Men	42 (42.8)	525 (44.6)	
Sexual orientation			
Heterosexual	85 (88.5)	1053 (89.5)	.76
Gay, lesbian, bisexual, or other	11 (11.5)	123 (10.5)	
Race and ethnicity			
Underrepresented in medicine^b^	17 (17.7)	224 (19.0)	.75
Non-underrepresented in medicine^c^	79 (82.3)	953 (81.0)	
Age, median (IQR), y	27 (26.0-29.8)	27 (26.0-29.0)	.04
Academic characteristics			
Specialty			
Surgical specialties^d^	20 (20.8)	202 (17.4)	.36
Nonsurgical specialties^e^	76 (79.2)	975 (82.8)	
Residency institution type			
University	78 (82.1)	1026 (88.0)	.25
Community	16 (16.8)	131 (11.2)	
Military	1 (1.1)	9 (0.8)	
Disability-related characteristics			
Type of disability		NA	NA
ADHD	65 (67.7)	NA	NA
Chronic health conditions	14 (14.6)	NA	NA
Deaf or hard of hearing	5 (5.2)	NA	NA
Learning disability	4 (4.2)	NA	NA
Mobility disability	1 (1.0)	NA	NA
Psychological disability^f^	4 (4.2)	NA	NA
Visual disability	3 (3.1)	NA	NA
Accommodation status		NA	NA
Residency provided accommodation	31 (32.3)	NA	NA
I have not requested because I feel I do not need accommodation	51 (53.1)	NA	NA
My request for accommodations was denied	1 (1.0)	NA	NA
My request for accommodation is under review	0	NA	NA
I have not requested accommodation for other reasons	13 (13.5)	NA	NA

Abbreviations: ADHD, attention-deficit/hyperactivity disorder; NA, not applicable.

^a^ χ^2^ test (categorical variables) or Independent Samples Mann-Whitney test (age).

^b^ Underrepresented in medicine (this group included residents self-identifying as African American, Latino, American Indian and Alaskan Native, and Pacific Islander).

^c^ Non-underrepresented in medicine (this group included residents self-identifying as Asian or White).

^d^ Surgical specialties included general surgery, obstetrics/gynecology, ophthalmology (integrated), and otolaryngology (head and neck surgery).

^e^ Nonsurgical specialties included anesthesiology, diagnostic radiology, integrated child neurology, dermatology, emergency medicine, family medicine, internal medicine, internal medicine/emergency medicine, internal medicine/pediatrics, internal medicine/psychiatry, neurology, pediatrics, pediatrics/emergency medicine, pediatrics/medical genetics and genomic, physical medicine and rehabilitation, psychiatry, psychiatry/family medicine, and transitional year.

^f^ Psychological disabilities include common mental illnesses for example: anxiety, depression, bipolar disorder, and obsessive-compulsive disorder.

RWD who self-reported that their program access needs were not met demonstrated a statistically significantly greater increase in depressive symptoms compared with nondisabled residents (median [IQR] survey score increase: with disabilities and without access needs met, 4.5 [1.0-10.5] vs without disabilities, 2.0 [0-4.0]; Dunn-Bonferroni-test for pairwise comparison, *z* = 2.5; adjusted *P* = .04) and were also significantly more likely to self-report major medical errors compared with nondisabled residents and RWD whose access needs were met (42.9% [6 respondents] vs without disabilities, 13.9% [163 respondents]; χ^2^ = 9.6; *P* = .008) (Table 2). No statistically significant differences in depressive symptoms or self-reported medical errors were observed between RWD with program access needs met and nondisabled residents (Table 2).

**Table 2.** Program Access, Increase in Depressive Symptoms, and Self-reported Medical Errors

Variable	Residents, No. (%)			*P* value^a^
	Without disabilities (n = 1177)	With disabilities and met program access needs (n = 82)	With disabilities and without met program access needs (n = 14)	
Increase in PHQ-9 depressive symptoms from baseline to 12-mo survey, median (IQR)	2.0 (0-4.0)^b^	2.0 (0-5.6)	4.5 (1.0-10.5)^b^	.02
Self-reported major medical error in the last 3 mo	163 (13.9)	11 (13.4)	6 (42.9)^c^	.008

Abbreviation: PHQ-9, Patient Health Questionnaire-9.

^a^ *P* values for χ^2^ tests (categorical variables) and Kruskal-Wallis (continuous variables). Dunn-Bonferroni-test, *z* = 2.5; adjusted *P* = .04.

^b^ Statistically significant difference between groups.

^c^ Statistically significant difference from other groups.

## Discussion

To our knowledge, this is the first study to examine the prevalence of disability among residents across multiple specialties, estimating a prevalence of 7.5%. Our study establishes an association between a lack of accessibility and heightened risk for depression and self-reported medical errors during training. This has important implications for the mental health and retention of disabled trainees, as well as for patient care.

Limitations include potential sampling and response bias (eg, underreporting or overreporting of disability, not understanding disability in the context of the Americans with Disabilities Act Amendments Act). Our findings, coupled with recent data showing low compliance with ACGME disability regulations,^6^ underscore the need to prioritize and enforce GME policies that improve access to training for disabled residents,^2^ both for the mental health of RWD and for the patients that they treat. Future research should focus on culture and climate that inform disability disclosure and accommodation requests in residency, including fear of stigma and bias.

## Notes

Supplement. **eMethods. **Survey questions used in the present study
