# Supplementary material for: Program Access, Depressive Symptoms, and Medical Errors Among Resident Physicians With Disability
Source: JAMA Netw Open. 2021 Dec 30;4(12):e2141511. doi: 10.1001/jamanetworkopen.2021.41511 (PMC8719234; doi:10.1001/jamanetworkopen.2021.41511)
Supplement: Supplement. — eMethods. Survey questions used in the present study [file jamanetwopen-e2141511-s001.pdf]

Supplemental Online Content

Meeks LM, Pereira-Lima K, Frank E, Stergiopoulos E, Ross KET, Sen S. Program access, depressive symptoms, and medical errors among resident physicians with disability. *JAMA Netw Open.* 2021;4(12):e2141511. doi:10.1001/jamanetworkopen.2021.41511

eMethods. Survey questions used in the present study

This supplemental material has been provided by the authors to give readers additional information about their work.

© 2021 Meeks LM et al. *JAMA Network Open.*

eMethods. Survey questions used in the present study

Baseline survey questions used in the present study

Demographic questions

- Date of Birth (MM/DD/YYYY) _______________

- Sex assigned at birth
  ( ) Male
  ( ) Female

- Which of the following best describes your sexual orientation?
  ( ) Heterosexual
  ( ) Gay/Lesbian
  ( ) Bisexual
  ( ) Other ______________
  ( ) Prefer not to say

- Ethnicity (check all that apply)
  ( ) Arab/Middle Eastern
  ( ) Asian
  ( ) Black/African American
  ( ) Latino/Hispanic
  ( ) Native American
  ( ) Pacific Islander
  ( ) White
  ( ) Other _______________

Patient Health Questionnaire-9 (PHQ-9)

© 2021 Meeks LM et al. *JAMA Network Open.*

MOOD SYMPTOMS For each statement, please mark the response which best represents how often you have been bothered by any of the following problems over the PAST 2 WEEKS

	Not at all (0)	Less than half the days (1)	More than half the days (2)	Nearly everyday (3)
Little interest or pleasure in doing things (1)	❍	❍	❍	❍
Feeling down, depressed or hopeless (2)	❍	❍	❍	❍
Trouble falling asleep, staying asleep or sleeping too much (3)	❍	❍	❍	❍
Feeling tired or having little energy (4)	❍	❍	❍	❍
Poor appetite or overeating (5)	❍	❍	❍	❍
Feeling badly about yourself, or that you are a failure, or that you have let yourself or your family down (6)	❍	❍	❍	❍
Trouble concentrating on things such as reading the newspaper or watching TV (7)	❍	❍	❍	❍
Moving or speaking so slow that others could have noticed or the opposite, being so fidgety or restless that you have been moving around a lot more than usual (8)	❍	❍	❍	❍
Thoughts that you would be better off dead or hurting yourself in some way (9)	❍	❍	❍	❍

© 2021 Meeks LM et al. *JAMA Network Open.*

End-of-year (12-month of internship) survey questions used in the present study

Disability-related questions

- Are you a person with a disability (e.g., ADHD, learning, psychological, chronic health, mobility, hearing, vision, etc.)?
  ( ) Yes
  ( ) No
  ( ) I do not know

- Which of the following best describes your disability?
  ( ) Attention deficit/hyperactivity disorder
  ( ) Chronic Health Disability
  ( ) Deaf or hard of hearing
  ( ) Learning disability
  ( ) Mobility disability
  ( ) Psychological disability
  ( ) Visual disability
  ( ) Other

- Has your residency program provided accommodations for your disability?
  ( ) Yes
  ( ) No

- Which of the following best describes why your medical school did not or has not provided accommodations:
  ( ) My request for accommodations was denied
  ( ) My request for accommodations is under review
  ( ) I have not requested accommodations because I feel I do not need accommodations
  ( ) I have not requested accommodations for other reasons

Medical errors

- Are you concerned you have made any major medical errors in the LAST 3 MONTHS?
  ( ) Yes
  ( ) No

Patient Health Questionnaire-9 (PHQ-9)

© 2021 Meeks LM et al. *JAMA Network Open.*

MOOD SYMPTOMS For each statement, please mark the response which best represents how often you have been bothered by any of the following problems over the PAST 2 WEEKS

	Not at all (0)	Less than half the days (1)	More than half the days (2)	Nearly everyday (3)
Little interest or pleasure in doing things (1)	❍	❍	❍	❍
Feeling down, depressed or hopeless (2)	❍	❍	❍	❍
Trouble falling asleep, staying asleep or sleeping too much (3)	❍	❍	❍	❍
Feeling tired or having little energy (4)	❍	❍	❍	❍
Poor appetite or overeating (5)	❍	❍	❍	❍
Feeling badly about yourself, or that you are a failure, or that you have let yourself or your family down (6)	❍	❍	❍	❍
Trouble concentrating on things such as reading the newspaper or watching TV (7)	❍	❍	❍	❍
Moving or speaking so slow that others could have noticed or the opposite, being so fidgety or restless that you have been moving around a lot more than usual (8)	❍	❍	❍	❍
Thoughts that you would be better off dead or hurting yourself in some way (9)	❍	❍	❍	❍

© 2021 Meeks LM et al. *JAMA Network Open.*
